# Food Insecurity Is Associated With Depressive Symptoms Among Brazilian Community Older Adults

**DOI:** 10.1093/geroni/igaa057.775

**Published:** 2020-12-16

**Authors:** Carolina Freiria, Graziele Silva, Larissa Hara, André Fattori, Flavia Borim, Tábatta Brito, Ligiana Corona

**Affiliations:** 1 University of Campinas, Campinas, Sao Paulo, Brazil; 2 University of Campinas, Limeira, Sao Paulo, Brazil; 3 University of Campinas, Campinas, Brazil; 4 Federal University of Alfenas, Alfenas, Minas Gerais, Brazil

## Abstract

Food security can be defined as when the individual has access to food consumption in adequate quality and quantity, respecting aspects such as age, physiological condition and cultural habits. While international studies showed the association of Food Insecurity (FI) and many negative health outcomes, like depressive symptoms, less is known about food insecurity among older people in Brazil, especially about its association with health. The aim of this study is to analyze the relationship between FI and Depressive Symptoms (DS) among community older Brazilian adults. Were included in this study 493 community older people with 60+. Geriatric Depression Scale were used to measure DS and for assessment of FI was used the short version of the Brazilian Food Insecurity Scale, added with one question involving functional limitations to buy food. Logistic regression was used to estimate the odds ratio (OR) adjusted for covariates (e.g., sex, education, age and familiar income). The prevalence of FI were 42.4% and the prevalence of DS were 71.5% of population. The prevalence of DS was higher in the group with FI than among those without F (78.9% vs 65.8% respectively; p=0.001). In the adjusted regression analysis, the chance of presenting positive symptomatology for depression was 1.87 times higher among the older people with FI (CI 1.18 –2.91; p=0.007). The findings demonstrate high prevalence of FI and DS indicating the importance of FI screening among community-based older people in order to avoid possible negative health outcomes in this population, such as the development of depressive symptoms.

